# The duration of lithium use and biological ageing: telomere length, frailty, metabolomic age and all-cause mortality

**DOI:** 10.1007/s11357-024-01142-y

**Published:** 2024-03-28

**Authors:** Julian Mutz, Win Lee Edwin Wong, Timothy R. Powell, Allan H. Young, Gavin S. Dawe, Cathryn M. Lewis

**Affiliations:** 1 Social, Genetic and Developmental Psychiatry Centre, Institute of Psychiatry, Psychology & Neuroscience, King’s College London, Memory Lane, London, UK; 2 Department of Pharmacology, Yong Loo Lin School of Medicine, National University of Singapore, Singapore, Singapore; 3 Healthy Longevity Translational Research Programme, Yong Loo Lin School of Medicine, National University of Singapore, Singapore, Singapore; 4 Department of Psychological Medicine, Institute of Psychiatry, Psychology & Neuroscience, King’s College London, London, UK; 5 South London & Maudsley NHS Foundation Trust, Bethlem Royal Hospital, Monks Orchard Road, London, UK; 6 Neurobiology Programme, Life Sciences Institute, National University of Singapore, Singapore, Singapore; 7 Department of Medical and Molecular Genetics, Faculty of Life Sciences and Medicine, King’s College London, London, UK

**Keywords:** Biological ageing, Lithium, Telomere, Frailty, Metabolomic age, Mortality

## Abstract

Lithium is an established first-line treatment for bipolar disorder. Beyond its therapeutic effect as a mood stabiliser, lithium exhibits potential anti-ageing effects. This study aimed to examine the relationship between the duration of lithium use, biological ageing and mortality. The UK Biobank is an observational study of middle-aged and older adults. We tested associations between the duration of lithium use (number of prescriptions, total duration of use and duration of the first prescription period) and telomere length, frailty, metabolomic age (MileAge) delta, pulse rate and all-cause mortality. Five hundred ninety-one individuals (mean age = 57.49 years; 55% females) had been prescribed lithium. There was no evidence that the number of prescriptions (*β* =  − 0.022, 95% CI − 0.081 to 0.037, *p* = 0.47), the total duration of use (*β* =  − 0.005, 95% CI − 0.023 to 0.013, *p* = 0.57) or the duration of the first prescription period (*β* =  − 0.018, 95% CI − 0.051 to 0.015, *p* = 0.29) correlated with telomere length. There was also no evidence that the duration of lithium use correlated with frailty or MileAge delta. However, a higher prescription count and a longer duration of use was associated with a lower pulse rate. The duration of lithium use did not predict all-cause mortality. We observed no evidence of associations between the duration of lithium use and biological ageing markers, including telomere length. Our findings suggest that the potential anti-ageing effects of lithium do not differ by the duration of use.

**Supplementary Information:**

The online version contains supplementary material available at 10.1007/s11357-024-01142-y.

## Introduction

Lithium is considered the gold standard treatment for patients with bipolar disorder who experience recurrent episodes of (hypo)mania and depression [1]. Lithium is also used to augment antidepressant medication, for example in patients with difficult-to-treat major depressive disorder [2]. While the evidence supports lithium’s therapeutic effects [3], its mechanisms of action are incompletely understood. Studies indicate that lithium influences neurotransmitter signalling and acts on second messenger systems including the adenyl cyclase and protein kinase A pathways [4].

Beyond its therapeutic effects as a mood stabiliser, multiple lines of evidence suggest that lithium may impact biological ageing and longevity [5]. For example, mitochondrial dysfunction is a hallmark of ageing [6], and lithium treatment has been shown to enhance mitochondrial metabolism by increasing the activity of electron transport complexes [7, 8]. Preclinical and clinical studies suggest that lithium can reduce oxidative stress, which is linked to several age-related diseases [9, 10]. Notably, studies have demonstrated that lithium extends the lifespan of model organisms, including *Caenorhabditis elegans* (“*C. elegans*”) and *Drosophila* [11, 12].

A focus of research on lithium’s potential anti-ageing properties has been to examine its link with telomere length. Telomeres, which are repetitive nucleoprotein complexes at the ends of chromosomes, progressively shorten with cell division and play an important role in maintaining genomic stability. Telomere attrition is a hallmark of ageing and is linked to cellular senescence [6]. Telomere dysfunction plays a role in many human diseases [13]. For example, meta-analyses suggest that individuals with bipolar disorder have shorter telomeres than healthy controls [14–16]. However, several studies have found no evidence of a difference in telomere length or observed longer telomeres in patients with bipolar disorder. These inconsistencies may in part result from differences in lithium treatment. Some clinical studies suggest that lithium protects against telomere attrition. Indeed, studies have shown that bipolar disorder patients treated with lithium had longer telomeres than healthy controls [17] and that lithium-treated patients had longer telomeres than patients who were not treated with lithium [17, 18]. While these group differences in telomere length could be due to other factors, several studies found that a longer duration of lithium treatment correlated with longer telomeres [19].

Building on studies in the UK Biobank that have identified predictors and correlates of telomere length [20–22], we examined the potential impact of the duration of lithium treatment on telomere length and other markers of biological ageing. We defined three measures of lithium treatment duration using primary care prescription records and assessed multiple markers of biological ageing and longevity, including telomere length, frailty, metabolomic age (MileAge) delta and all-cause mortality. The overall aim was to elucidate the broader implications of lithium treatment on biological ageing.

## Methods

### Study population

The UK Biobank is a prospective community-based study that recruited over 500,000 middle-aged and older adults, aged 40 to 69 years [23]. Individuals who were registered with the UK National Health Service (NHS) and lived within a 25-mi (~ 40 km) radius of one of 22 assessment centres were invited to participate. At the baseline assessment, which took place between 2006 and 2010, participants reported their sociodemographic characteristics, health behaviours and medical history. They also underwent physical examinations and had blood and urine samples taken. Data linkage to primary care health records is available for about 230,000 participants.

### Duration of lithium use

The duration of lithium use was derived from medication prescriptions recorded by general practitioner practices as part of routine patient care. These data included prescriptions recorded between July 1945 and September 2017 and were available for 222,073 participants. Lithium prescriptions were identified using a combination of the British National Formulary (BNF), Read v2 and Dictionary of Medicines and Devices (dm + d) codes and by searching for relevant string permutations across the prescription medication names. The duration of lithium use was defined in three ways, similar to a previous study on antidepressants [24]. First, we calculated the total number of lithium prescriptions across all primary care prescription records. Second, we calculated the total number of weeks across all prescription windows, which we defined as periods of less than 90 days between consecutive prescriptions. Third, we calculated the number of weeks of the first prescription window. Prescriptions after the baseline assessment date were excluded from these calculations.

### Telomere length

Leukocyte telomere length was measured using a quantitative polymerase chain reaction (PCR) assay that expresses telomere length as the ratio of the telomere repeat copy number (T) relative to a single-copy gene (S) that encodes haemoglobin subunit beta [25]. The T/S ratio is proportional to an individual’s average telomere length [26]. Measurements were adjusted for operational and technical parameters (PCR machine, staff member, enzyme batch, primer batch, temperature, humidity, primer batch × PCR machine, primer batch × staff member, A260/A280 ratio of the DNA sample and A260/A280 ratio squared), log_*e*_ transformed and *Z*-standardised.

### Frailty Index

A frailty Index was derived from health deficits that met the following criteria: indicators of poor health, more prevalent in older individuals, neither rare nor universal, covering multiple areas of functioning and available for ≥ 80% of participants [27]. The index included 49 variables ascertained via touch-screen questionnaires and nurse-led interviews, including cardiometabolic, cranial, immunological, musculoskeletal, respiratory and sensory traits, well-being, infirmity, cancer and pain. Categorical variables were dichotomised (no deficit = 0; deficit = 1), and ordinal variables were mapped onto a score between 0 and 1. The sum of deficits was divided by the total number of possible deficits, resulting in frailty Index scores between 0 and 1, with higher scores indicating greater levels of frailty [28]. Participants with missing data for ≥ 10/49 variables were excluded [27].

### Metabolomic age (MileAge) delta

Nuclear magnetic resonance (NMR) spectroscopy–derived metabolomic biomarkers were quantified in non-fasting plasma samples using the Nightingale Health platform, which ascertains 168 circulating metabolites using a high-throughput standardised protocol [29]. In 101,359 participants, we developed a metabolomic ageing clock using a Cubist rule–based regression model [30]. This algorithm is an ensemble technique that derives rules from decision trees and fits linear regression models in the subsets of data defined by these rules. The model incorporates boosting techniques and may adjust predictions based on *k*-nearest neighbours [31, 32]. Model performance was assessed using nested cross-validation with 10 outer loops and 5 inner folds for hyperparameter tuning. The cross-validation mean absolute error (MAE) was 5.42 years. Metabolomic age (MileAge) delta represents the difference between predicted and chronological age, with positive and negative values indicating accelerated and decelerated biological ageing, respectively [30].

### Pulse rate

Resting pulse rate in beats per minute was recorded using an Omron 705 IT digital blood pressure monitor device or, exceptionally, a manual sphygmomanometer. We calculated the average of the two available readings to reduce potential measurement error.

### All-cause mortality

The date of death was obtained through linkage with national death registries: NHS Digital (England and Wales) and the NHS Central Register (Scotland). The censoring date was 30 November 2022. The most recent death was recorded on 19 December 2022, although the data were incomplete for December 2022.

### Covariates

Covariates for the cross-sectional and prospective analyses were identified from prior studies and domain knowledge. They included chronological age, sex, body mass index (kg/m^2^), morbidity count (none/one, two, three, four or five +), gross annual household income (< £18,000, £18,000–£30,999, £31,000–£51,999 or £52,000–£100,000/ > £100,000), highest educational/professional qualification (none, O levels/GCSEs/CSEs, A levels/NVQ/HND/HNC or degree) and cohabitation status (single or with spouse/partner). A directed acyclic graph (DAG) of the relationships between the duration of lithium use, telomere length and selected confounders is shown in Figure S1.

### Statistical analyses

The data processing and analyses were performed in R (version 4.2). Sample characteristics were summarised using means and standard deviations or counts and percentages. Associations between the duration of lithium use (number of prescriptions, total duration of use and duration of the first prescription period) and telomere length were estimated using ordinary least squares regression. We further tested associations between the duration of lithium use and two other indicators of biological age (the frailty Index and MileAge delta) and pulse rate. Hazard ratios (HRs) and 95% confidence intervals were estimated using Cox proportional hazards models to examine associations between the duration of lithium use and all-cause mortality. We examined both quintiles of the distribution, with the lowest quintile as the reference group, and spline functions of the distribution with the median as reference value. Age in years was used as the underlying time axis, with age 40 as the start of follow-up. The cross-sectional and prospective analyses were adjusted for chronological age and sex (model 1) and chronological age, sex, body mass index, morbidity count, household income, highest qualification and cohabitation status (model 2).

### Sensitivity analyses

We conducted two sensitivity analyses of the duration of lithium use and biological ageing. First, we restricted the sample to individuals with a total duration of lithium use of (a) at least 1 year (52 weeks) or (b) at least 4.5 years (234 weeks) because of prior research identifying an association between the duration of lithium use and telomere length only in chronic lithium users [19]. Second, we additionally adjusted for current antidepressant medication use (yes/no) at the time of the baseline assessment because of prior evidence that antidepressant medication use may be associated with shorter telomeres [20, 33, 34], though not all studies support this finding [35, 36]. We did not adjust for the duration of antidepressant use because previous studies found no evidence that telomere length differed between individuals who had been on antidepressant medications for periods shorter or longer than 6 months [37] or that telomere length was associated with the lifetime duration of antidepressant use [38].

## Results

### Sample characteristics

Of the 502,476 participants in the UK Biobank, 222,073 individuals (about 44.2% of the sample) had prescription data available through their linked primary care records. Of the 773 participants with a lithium prescription in their primary care record, *n* = 182 were excluded because their first prescription followed the baseline assessment. The duration of lithium use was estimated for 591 participants (Figure S2). Sample characteristics are shown in Table 1. Based on our previously reported criteria [39, 40], 71.74% of participants had a lifetime history of mood disorders (*n* = 248 had bipolar disorder and *n* = 176 had depression).

**Table 1:** Sample characteristics

	Lithium duration (*N* = 591)	Primary care (*N* = 221,482)	No primary care^1^ (*N* = 280,403)
Age; mean (SD)	57.49 (7.51)	56.58 (8.05)	56.48 (8.13)
Sex			
Female	327 (55.3%)	121,618 (54.9%)	151,420 (54.0%)
Male	264 (44.7%)	99,864 (45.1%)	128,982 (46.0%)
Ethnicity			
White	572 (96.8%)	210,119 (94.9%)	261,977 (93.4%)
Non-White	14 (2.4%)	10,322 (4.7%)	16,695 (6.0%)
Missing^2^	5 (0.8%)	1041 (0.5%)	1731 (0.6%)
Highest qualification			
None	119 (20.1%)	39,882 (18.0%)	45,265 (16.1%)
O levels/GCSEs/CSEs	125 (21.2%)	58,292 (26.3%)	73,662 (26.3%)
A levels/NVQ/HND/HNC^3^	118 (20.0%)	50,521 (22.8%)	63,207 (22.5%)
Degree	222 (37.6%)	69,916 (31.6%)	91,014 (32.5%)
Missing^2^	7 (1.2%)	2871 (1.3%)	7255 (2.6%)
Household income^4^			
Very low	198 (33.5%)	45,278 (20.4%)	51,717 (18.4%)
Low	132 (22.3%)	49,247 (22.2%)	58,794 (21.0%)
Medium	95 (16.1%)	49,037 (22.1%)	61,637 (22.0%)
High/very high	72 (12.2%)	45,305 (20.5%)	63,809 (22.8%)
Missing^2^	94 (15.9%)	32,615 (14.7%)	44,446 (15.9%)
Cohabitation			
With partner	359 (60.7%)	160,735 (72.6%)	201,961 (72.0%)
Single	55 (9.3%)	17,885 (8.1%)	24,172 (8.6%)
Missing^2^	177 (29.9%)	42,862 (19.4%)	54,270 (19.4%)
Smoking status			
Never	293 (49.6%)	121,023 (54.6%)	152,188 (54.3%)
Former	186 (31.5%)	76,153 (34.4%)	96,711 (34.5%)
Current	106 (17.9%)	23,143 (10.4%)	29,724 (10.6%)
Missing^2^	6 (1.0%)	1163 (0.5%)	1780 (0.6%)
Morbidity count			
None/one	119 (20.1%)	108,494 (49.0%)	140,930 (50.3%)
Two	150 (25.4%)	44,962 (20.3%)	55,724 (19.9%)
Three	116 (19.6%)	29,465 (13.3%)	36,037 (12.9%)
Four	77 (13.0%)	17,297 (7.8%)	20,903 (7.5%)
Five +	126 (21.3%)	20,903 (9.4%)	26,310 (9.4%)
Missing^2^	3 (0.5%)	361 (0.2%)	499 (0.2%)
Baseline lithium Rx^5^			
No	296 (50.1%)	204,499 (92.3%)	252,446 (90.0%)
Yes	261 (44.2%)	83 (0.0%)	417 (0.1%)
Missing^2^	34 (5.8%)	16,900 (7.6%)	27,540 (9.8%)
Baseline cholesterol-lowering Rx^5^			
No	425 (71.9%)	171,354 (77.4%)	217,473 (77.6%)
Yes	161 (27.2%)	48,000 (21.7%)	57,335 (20.4%)
Missing^2^	5 (0.8%)	2128 (1.0%)	5595 (2.0%)
Fasting time			
Less than 8 h	563 (95.3%)	211,768 (95.6%)	267,565 (95.4%)
At least 8 h	24 (4.1%)	9179 (4.1%)	12,143 (4.3%)
Missing^2^	4 (0.7%)	535 (0.2%)	695 (0.2%)
Body mass index; mean (SD)^6^	28.70 (5.34)	27.54 (4.83)	27.34 (4.78)

Numbers shown are counts and percentages unless indicated otherwise

*SD* standard deviation, *GCSEs* general certificate of secondary education, *CSE* certificate of secondary education, *NVQ* national vocational qualification, *HND* higher national diploma, *HNC* higher national certificate, *Rx* prescription

^1^Information on age and sex was missing for one participant in the sample without linked primary care data

^2^Missing data also includes “do not know”, “prefer not to answer” or “not applicable”

^3^Also includes ‘other professional qualifications’

^4^Annual household income groups: very low (< £18,000), low (£18,000–£30,999), middle (£31,000–£51,999), high (£52,000–£100,000) and very high (> £100,000)

^5^Prescriptions refer to *current* medication use at the time of the baseline assessment

^6^Missing data for body mass index: *n* = 10, *n* = 1344 and *n* = 1751

### Duration of lithium use and telomere length

**Fig. 1:**
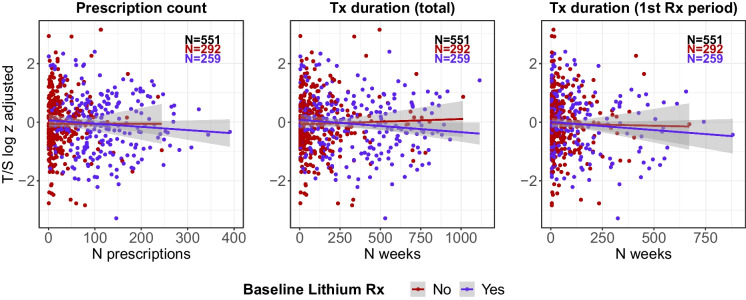
Scatter plots of telomere length (T/S ratio log_*e*_ transformed and *Z*-standardised) by lithium prescription count, total duration of use and duration of the first prescription period. The lines were estimated using ordinary least squares regression, and the shaded areas correspond to 95% confidence intervals. Lithium use (yes/no) at the time of the baseline assessment when telomere length was measured is shown in different colours. Individuals without baseline data on current lithium use are not shown. Tx = treatment; Rx = prescription

In the model adjusted for chronological age and sex, there was no evidence that the number of lithium prescriptions (*N* = 569, *β* =  − 0.022, 95% CI − 0.081 to 0.037, *p* = 0.47), the total duration of use (*N* = 569, *β* =  − 0.005, 95% CI − 0.023 to 0.013, *p* = 0.57) or the duration of the first prescription period (*N* = 569, *β* =  − 0.018, 95% CI − 0.051 to 0.015, *p* = 0.29) were associated with telomere length. Additional adjustments for body mass index, morbidity count, household income, highest qualification and cohabitation status had no discernible impact on these results (Table 2). Individuals with a higher prescription count or longer duration of lithium use were more likely to use lithium at the time of the baseline assessment (Fig. 1).

**Table 2:** Associations between the duration of lithium use and biological ageing

	Model 1 (adj. for age and sex)					Model 2 (full adjustment)				
	*n*	Beta	95% CI		*p*	*n*	Beta	95% CI		*p*
Telomere length										
Rx count	569	− 0.022	− 0.081	0.037	0.47	567	− 0.017	− 0.076	0.043	0.58
Total duration	569	− 0.005	− 0.023	0.013	0.57	567	− 0.004	− 0.022	0.015	0.71
Duration first Rx	569	− 0.018	− 0.051	0.015	0.29	567	− 0.014	− 0.048	0.019	0.40
Frailty index										
Rx count	585	− 0.045	− 0.104	0.014	0.13	580	− 0.043	− 0.091	0.004	0.08
Total duration	585	− 0.012	− 0.030	0.006	0.21	580	− 0.013	− 0.028	0.001	0.07
Duration first Rx	585	− 0.027	− 0.060	0.007	0.12	580	− 0.021	− 0.048	0.007	0.14
Metabolomic age delta										
Rx count	120	− 0.036	− 0.590	0.518	0.90	120	− 0.022	− 0.583	0.540	0.94
Total duration	120	0.030	− 0.133	0.194	0.72	120	− 0.009	− 0.180	0.161	0.91
Duration first Rx	120	0.248	− 0.079	0.576	0.14	120	0.208	− 0.128	0.543	0.22
Pulse rate										
Rx count	586	− 0.061	− 0.119	− 0.002	0.04	583	− 0.058	− 0.116	0.0001	0.05
Total duration	586	− 0.026	− 0.044	− 0.008	0.005	583	− 0.024	− 0.042	− 0.006	0.01
Duration first Rx	586	− 0.021	− 0.055	0.012	0.21	583	− 0.022	− 0.056	0.011	0.18

The measurement units have been rescaled. The number of prescriptions was analysed in units of 50 prescriptions; the total duration of use and the duration of the first prescription period were expressed in years. Model 1 was adjusted for age and sex; model 2 was adjusted for age, sex, body mass index, morbidity count, household income, highest qualification and cohabitation status

*Rx* prescription, *CI* confidence interval

### Duration of lithium use, frailty and metabolomic age (MileAge) delta

**Fig. 2:**
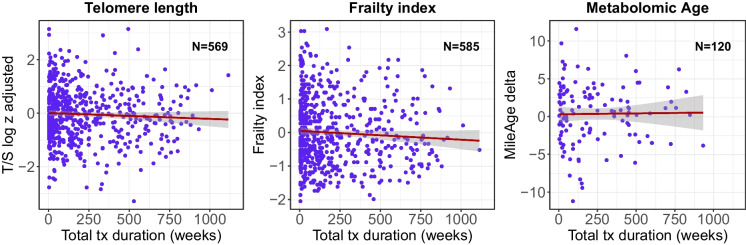
Scatter plots of telomere length (T/S ratio log_*e*_ transformed and *Z*-standardised), the frailty Index and metabolomic age (MileAge) delta by total duration of lithium use. The lines were estimated using ordinary least squares regression, and the shaded areas correspond to 95% confidence intervals. tx = treatment

There was no evidence that the duration of lithium use was associated with the frailty Index (*N* = 585, *β* =  − 0.012, 95% CI − 0.030 to 0.006, *p* = 0.21) or MileAge delta (*N* = 120, *β* = 0.030, 95% CI − 0.133 to 0.194, *p* = 0.72) (Fig. 2). This finding was consistent across all measures of the duration of lithium use and adjustment for additional covariates (Table 2).

### Duration of lithium use and pulse rate

To ensure that the lack of statistically significant associations was not due to how we defined the duration of lithium use, we further tested associations between the duration of lithium use and pulse rate (Figure S3). The number of lithium prescriptions (*N* = 586, *β* =  − 0.061, 95% CI − 0.119 to − 0.002, *p* = 0.04) and the total duration of lithium use (*N* = 586, *β* =  − 0.026, 95% CI − 0.044 to − 0.008, *p* = 0.005) were both associated with a lower pulse rate (Table 2).

### Duration of lithium use and all-cause mortality

**Fig. 3:**
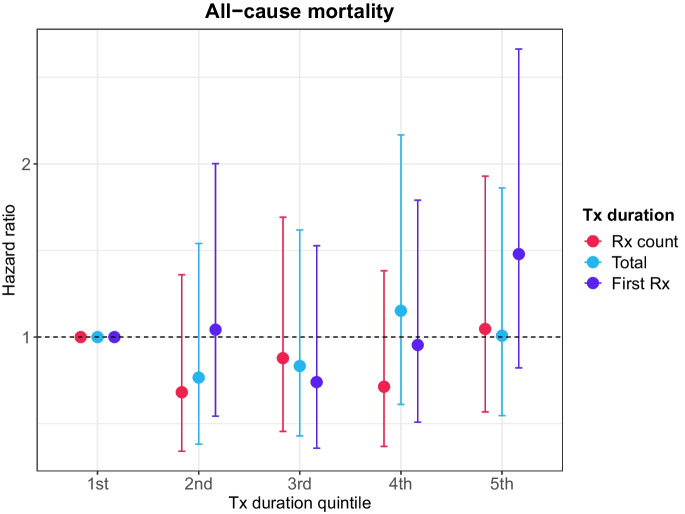
Hazard ratios (HR) and 95% confidence intervals from Cox proportional hazards models for all-cause mortality. Age (in years) was used as the underlying time axis. The model was adjusted for age and sex. Reference group: individuals in the lowest quintile of the distribution of the duration of lithium use (prescription count, total duration and duration of the first prescription period). Tx = treatment; Rx = prescription

The median duration of follow-up of censored individuals was 13.71 years (IQR = 1.35 years), with more than 7686 person-years of follow-up. During this period, 94 deaths were observed. There was no evidence that the duration of lithium use predicted all-cause mortality (Table 3). Comparing individuals in the second to fifth quintile of the distribution of the duration of lithium use to individuals in the first quintile resulted in no statistically significant differences in all-cause mortality (lowest *p* = 0.08) (Fig. 3).

**Table 3:** Associations between the duration of lithium use and all-cause mortality

		Model 1 (adj. for age and sex)						Model 2 (full adjustment)					
		*N*_total_	*N*_died_	HR	95% CI		*p*	*N*_total_	*N*_died_	HR	95% CI		*p*
Rx count													
1st	≤ 7	119	19	Reference				118	19	Reference			
2nd	8–23	118	16	0.68	0.34	1.36	0.28	116	15	0.53	0.26	1.09	0.08
3rd	24–60	118	19	0.88	0.46	1.69	0.70	117	19	0.74	0.37	1.48	0.40
4th	61–114	118	18	0.71	0.37	1.38	0.32	118	18	0.68	0.34	1.35	0.27
5th	≥ 115	118	25	1.05	0.57	1.93	0.88	116	25	0.91	0.48	1.74	0.79
Total duration													
1st	≤ 22	119	19	Reference				118	19	Reference			
2nd	23–83	118	14	0.77	0.38	1.54	0.45	117	13	0.61	0.29	1.26	0.18
3rd	84–187	118	19	0.83	0.43	1.62	0.59	115	19	0.74	0.37	1.49	0.40
4th	188–426	118	21	1.15	0.61	2.17	0.66	118	21	0.95	0.50	1.83	0.88
5th	≥ 427	118	24	1.01	0.55	1.86	0.98	117	24	0.91	0.48	1.73	0.78
Duration first Rx													
1st	≤ 4	119	21	Reference				117	20	Reference			
2nd	5–16	118	17	1.04	0.54	2.00	0.90	118	17	0.96	0.49	1.92	0.92
3rd	17–49	118	13	0.74	0.36	1.53	0.42	117	13	0.71	0.34	1.50	0.37
4th	50–130	118	19	0.95	0.51	1.79	0.88	117	19	1.05	0.54	2.03	0.90
5th	≥ 131	118	27	1.48	0.82	2.66	0.19	116	27	1.46	0.80	2.68	0.22

Hazard ratios and 95% confidence intervals from Cox proportional hazards models for all-cause mortality. Age (in years) was used as the underlying time axis. Model 1 was adjusted for age and sex; model 2 was adjusted for age, sex, body mass index, morbidity count, household income, highest qualification and cohabitation status

*Rx* prescription, *HR* hazard ratio, *CI* confidence interval

**Fig. 4:**
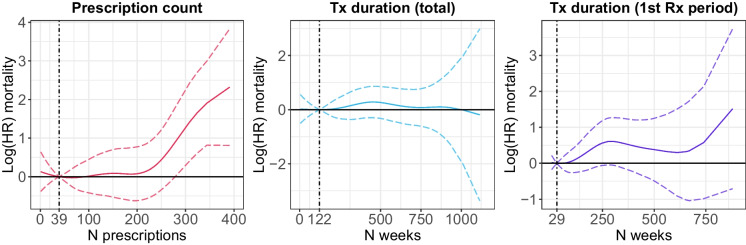
Log hazard ratios and 95% confidence intervals from Cox proportional hazards models for all-cause mortality. Age (in years) was used as the underlying time axis. The model was adjusted for age and sex. Vertical lines indicate the median of the distribution. Tx = treatment; Rx = prescription

Modelling the mortality hazard as a spline function of the duration of lithium use, instead of by quintiles of the distribution, provided little evidence of a statistically significant association between the duration of lithium use and all-cause mortality (Fig. 4 and Figure S4). There was, however, some evidence that prescription counts greater than about 300 were associated with a higher mortality risk.

### Sensitivity analyses

Excluding participants with a total duration of lithium use of less than 1 year or less than 4.5 years provided little evidence that the duration of lithium use was associated with biological ageing (Table S1 and Table S2). There was some evidence that a longer duration of the first lithium prescription period was associated with a higher MileAge delta, although this finding did not survive multiple testing corrections in the fully adjusted model. The duration of lithium use was not associated with pulse rate in these analyses.

The relationship between telomere length and the duration of lithium use stratified by current antidepressant use at the time of the baseline assessment is shown in Figure S5. Additional adjustments for current antidepressant use had no discernible impact on the analyses of the relationship between the duration of lithium use and biological ageing (Table S3). However, the association between a higher prescription count and a longer total duration of lithium use and pulse rate was stronger in these analyses.

## Discussion

We examined the relationship between the duration of lithium use and multiple markers of biological ageing, including telomere length, frailty, metabolomic age (MileAge) delta and all-cause mortality. Both cross-sectional and prospective analyses provided little evidence of an association between the duration of lithium use and biological ageing or mortality.

Our findings are inconsistent with most prior studies of the duration of lithium use and telomere length, several of which identified a positive association between lithium and telomere length. Martinsson et al. found that lithium-treated bipolar disorder patients had on average 35% longer telomeres than healthy controls [17]. In the same study, the duration of lithium treatment was positively correlated with telomere length, particularly in patients who had been treated for more than 2.5 years. Pisanu et al. [41] and Coutts et al. [19] corroborated these observations, identifying statistically significant associations between chronic lithium treatment and telomere length in patients with bipolar disorder. Squassina et al. also reported a positive correlation between the duration of lithium use and telomere length in 150 bipolar disorder patients who had been treated for more than 2 years [42]. A mechanistic in vitro study provided further evidence supporting lithium’s putative effect on telomeres by demonstrating that lithium administration to lymphoblastoid cell lines from bipolar disorder patients increased telomere length [18]. Nevertheless, there is some precedent for our findings. Ferensztajn-Rochowiak et al. reported that in a sample of 41 patients with bipolar disorder, the duration of lithium treatment did not correlate with telomere length in peripheral blood leukocytes [43]. Another in vitro study found that the administration of lithium to lymphoblastoid cell lines from bipolar disorder patients had no effect on telomere length [44], though it is worth noting that these patients had previously been treated with lithium.

There are several potential explanations for the observed differences between our findings and those of prior studies. For example, most clinical studies determined the duration of lithium treatment retrospectively during the study visit, while we derived the duration of lithium use in our study from prescription records. Furthermore, there is some evidence that clinical factors such as bipolar disorder diagnosis, number of episodes, chronicity and severity of illness impact telomere length. The participants in our study likely represent a healthier subset of the population than the patients included in prior clinical studies. Moreover, genetic factors can moderate the effect of lithium on telomere length [19]. Although we adjusted for several confounders in our analyses, we also found little evidence of any statistical associations in the model adjusted only for chronological age and sex. The sample size of our study (*n* = 569) is one of the largest in the field, and we used three definitions of the duration of lithium use to increase the robustness of our findings. In addition, we conducted a sensitivity analysis of pulse rate, which we expected to be negatively correlated with the duration of lithium use [45]. The observed correlation between the duration of lithium use and a lower pulse rate supports the validity of our measures of the duration of lithium use.

There has been little prior research on the relationship between lithium use and the other biological ageing markers explored in this study. Okazaki et al. found that bipolar disorder patients who were taking mood stabilisers had a younger epigenetic age, according to Horvath’s epigenetic ageing clock, than patients who were not taking mood stabilisers [46]. However, an in vitro study of lymphoblastoid cell lines did not find that administration of lithium impacted the Horvath clock [18]. We found no evidence of an association between the duration of lithium use and metabolomic age (MileAge) delta in our study. However, it is worth noting that epigenetic and metabolomic age may not strongly correlate [47]. We also explored the relationship between the duration of lithium use and frailty, which is a measure of age-related health deficit accumulation. While some evidence suggests that frailty and telomere length are not strongly linked [48], frailty is a key marker of biological ageing and physical ill-health [49, 50]. Our study provided no evidence of a statistically significant association between the duration of lithium use and frailty. Taken together, these findings suggest that the lack of association between the duration of lithium use and biological ageing—at least in our sample—was not specific to telomere length.

The *duration* of lithium use was not linked to all-cause mortality in our study. Multiple observational studies have previously shown that lithium use is associated with a lower mortality risk. For example, a cohort study of 826 patients in Finland found that lithium treatment, compared to no lithium treatment, was associated with a lower all-cause mortality risk [51]. Another cohort study in Taiwan found that lithium was associated with the lowest all-cause mortality risk in bipolar disorder patients, relative to other mood stabiliser treatments [52]. A recent study in the UK Biobank found that lithium treatment was associated with a lower all-cause mortality risk than treatment with antipsychotics [53]. However, it is important to note that we examined the duration of lithium use, not whether patients were treated versus not treated with lithium, which prior studies examined. As such, our findings suggest that there may not be a link between longer duration of lithium use and all-cause mortality in a community-based, real-world sample. These findings do not imply that lithium use (yes/no) is not linked to all-cause mortality, and they do not invalidate findings from clinical trials where lithium adherence is more controlled.

### Limitations

We acknowledge certain limitations to our study. The UK Biobank primarily recruited individuals between the ages of 40 and 69 years, limiting the conclusions drawn from our findings to middle-aged and older adults. There is evidence of a healthy volunteer participation bias in the UK Biobank [54, 55], which could mean that the participants in our study were healthier than the patients included in prior clinical studies. The observational nature of our study precludes any causal inferences, and residual confounding factors may exist despite our efforts to account for relevant covariates. The duration of lithium use was derived from primary care prescription records. Prescriptions may not perfectly reflect medication use, and there is some evidence that, despite the need for regular blood tests, adherence to lithium is poor in clinical practice [56]; furthermore, prescription dates are a proxy of date of use. Nonetheless, we derived and tested three different definitions of the duration of lithium use to be as robust as possible within these constraints. Telomere length was measured using leukocyte DNA, and findings might differ from telomere length measured in other tissues. However, prior evidence suggests that leukocyte telomere length correlates well with telomere length in other tissues [57]. Finally, we could only examine *average* telomere length and were unable to evaluate whether the duration of lithium use was associated with variability in telomere length, which may be more important for telomere dysfunction and biological ageing [26].

### Conclusion

Our findings, in a larger sample, challenge prior research on the relationship between the duration of lithium use and telomere length. Given that we also did not observe statistically significant associations with other biological ageing markers such as frailty and metabolomic age (MileAge) delta, nor with all-cause mortality, our findings suggest that the potential anti-ageing effects of lithium do not differ by the duration of use. Our findings encourage further scientific inquiry into the complex interplay between lithium medication, biological ageing and lifespan.

## Supplementary Information

Below is the link to the electronic supplementary material.

Supplementary file1 (DOCX 1097 KB)

## Data Availability

UK Biobank data used are available to all *bona fide* researchers for health-related research that is in the public interest, subject to an application process and approval criteria. Study materials are publicly available online at http://www.ukbiobank.ac.uk.
